# Investigation of Different Features for Baseline-Free RAPID Damage-Imaging Algorithm Using Guided Waves Applied to Metallic and Composite Plates

**DOI:** 10.3390/ma16237390

**Published:** 2023-11-28

**Authors:** Aadhik Asokkumar, Renaldas Raišutis, Dario J. Pasadas, Vykintas Samaitis, Liudas Mažeika

**Affiliations:** 1 Prof. K. Baršauskas Ultrasound Research Institute, Kaunas University of Technology, K. Baršausko St. 59, 51423 Kaunas, Lithuania; renaldas.raisutis@ktu.lt (R.R.); vykintas.samaitis@ktu.lt (V.S.); liudas.mazeika@ktu.lt (L.M.); 2 Instituto de Telecomunicações, Instituto Superior Técnico, Universidade de Lisboa, 1049-001 Lisbon, Portugal; dpasadas@lx.it.pt

**Keywords:** damage imaging, guided waves, structural health monitoring, RAPID, composites, baseline-free

## Abstract

In guided-wave-based damage-imaging algorithms, damage reconstruction typically involves comparing the signals with and without a defect. However, in many cases, defect-free data may not be available. Therefore, in this study, baseline and baseline-free approaches were used for damage imaging, exploiting not only the amplitude of the signal as the feature but also five additional features, namely, the amplitude of the sparse signal after deconvolution, the amplitude of the coefficients at the excitation frequency from the re-assigned short-time Fourier transform, the time of flight determined from cross-correlation, kurtosis in the time domain, and kurtosis in the frequency domain. For this study, three different plates with different types of defects were considered: a metallic plate with a notch-type artificial defect, a pultruded type of composite plate with an impact defect, and a laminate composite plate with plexiglass serving as an added mass damper artificial defect. The Reconstruction Algorithm for Probabilistic Inspection of Damage (the RAPID algorithm) was used to characterize the defects on the three plates, and the defect parameters were then quantified by creating an ellipse after thresholding.

## 1. Introduction

Structural Health Monitoring (SHM) plays a critical role in ensuring the integrity and safety of various large engineering structures, such as bridges, wind turbines, aerospace components, etc. Guided-wave-based damage-imaging methods offer a promising approach for performing active SHM on large structures like aircrafts. Guided waves are ultrasonic waves that propagate along thin plate-like structures, and they can efficiently interact with defects, enabling the detection and localization of damage over a large area.

Compared to conventional Non-Destructive Testing (NDT) methods, guided-wave-based imaging for SHM offers several advantages. Firstly, it provides a rapid and cost-effective inspection process while reducing downtime. Secondly, it allows for the detection of hidden defects that are not easily observable using traditional NDT methods. Additionally, guided-wave-based imaging enables the continuous monitoring of large structural areas during operation, facilitating early damage detection and preventive maintenance. One of the key challenges in SHM is the inherent complexity and uniqueness of each structure due to their different material properties, defect characteristics, and environmental conditions, rendering a one-size-fits-all approach ineffective.

Several damage-imaging algorithms have been proposed in the literature for damage evaluation, such as the delay-and-sum imaging method [1,2], the time reversal imaging method [3,4], and the RAPID imaging method [5]. The Reconstruction Algorithm for Probabilistic Inspection of Defects (RAPID) is a widely used approach in guided-wave-based defect reconstruction algorithms, generally used for damage detection and localization. As a damage index (feature), the conventional RAPID algorithm uses the Signal Difference Coefficient (SDC), which is based on the Pearson’s correlation coefficient between the signal with the defect and without the defect [6]. In summary, from the studies mentioned above involving the RAPID algorithm, the following important drawbacks can be observed:

- Reconstruction is performed with only one type of feature for damage reconstruction, i.e., the Pearson correlation coefficient;
- Reconstruction can be performed only when both the baseline data and defect data are available because the SDC functions by comparing the defect-free data and defect data;
- This algorithm is generally used for the localization of a defect, and it is generally sufficient in this task in most cases. But the quantification of the defect, such as with respect to the length, orientation, and size of the damage areas, is usually ignored, which limits the practical application of this algorithm.

Therefore, this research’s primary aim is to investigate and unlock the latent capabilities of the RAPID algorithm, seeking to address the identified limitations, which leads us to this study’s core objectives:

- Performing modifications to the RAPID algorithm to be able to perform damage imaging with only baseline-free data;
- Exploring features other than Pearson’s correlation coefficient that can be used for the RAPID algorithm;
- Devising a quantification method with which to determine the parameters of a defect.

Concerning the features, in total, six features were considered: the amplitude of the signal, the amplitude of the sparse signal after deconvolution, the amplitude of the coefficients at the excitation frequency obtained using the Re-assigned Short-Time Fourier Transform (RSTFT), Time of Flight (ToF) derived through cross-correlation, and kurtosis in the frequency and time domains. After applying the RAPID algorithm, some quantitative analyses were performed on the resulting images. Specifically, deconvolution was considered because it has been shown in various fields of study [7,8,9,10,11] to be useful for localizing the energy of a signal in the time domain and for increasing the signal-to-noise ratio (SNR). Also, the RSTFT was considered for the Time Frequency Representation (TFR) because it has been shown [12] to be one of the best TFRs when compared to continuous wavelet transform, Wigner Ville distribution, etc.

To assess the novel features of the RAPID algorithm, additional variables were introduced during the experimental phase, thereby enhancing the complexity of this study. This augmentation encompasses several aspects:

- The inclusion of three distinct specimen types;
- The introduction of varying defect types within each specimen;
- The deployment of diverse transducers to capture data for the different specimen scenarios, facilitating comprehensive damage imaging.

This paper is organized as follows: Section 2 describes the material properties and the experimental details for each plate. Section 3 describes the RAPID algorithm. In Section 4, the six features used in this study are detailed, with an example provided for each feature. Section 5 presents the results from the experiments conducted on the three different plates, including the quantification of defect parameters estimated after thresholding using the ellipse method. Finally, Section 6 concludes the paper by summarizing the findings, discussing the implications, and outlining potential avenues for future research.

## 2. Specimens Used in the Study

The first specimen is a metallic plate with a notch-type artificial defect, representing a commonly encountered surface and subsurface flaw in metallic aircraft structures. Contact-type angle wedge transducers were used to generate and measure the guided waves for this plate. The second plate is a pultruded composite plate with a 12 J impact defect, simulating the impact damage often observed in aerospace and automotive structures. Air-coupled transducers were used to generate and measure the guided waves for this plate. Lastly, a laminate composite plate with plexiglass was used as an added mass damper to simulate a defect in the third specimen; here, Macro Fiber Composite (MFC) transducers were used to generate guided waves. The chosen plates with their respective defects provide a representative sample of the challenges encountered in real-world structural health monitoring scenarios. Information on the specimens, material properties, transducers, and defect parameters are tabulated in Table 1. 

### 2.1. Aluminum Plate

**Figure 1:**
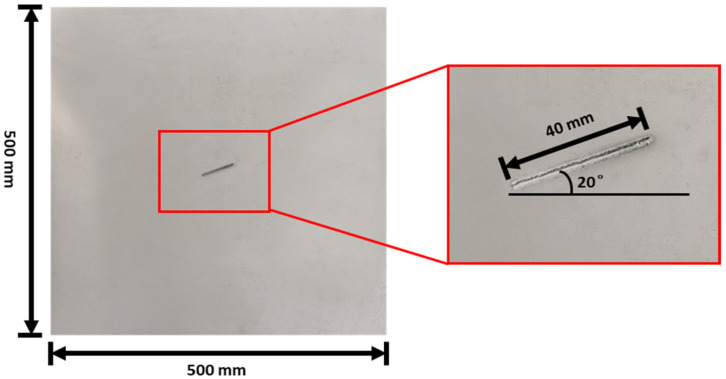
Aluminium-1050 plate along with the notch-type defect used for the study.

**Figure 2:**
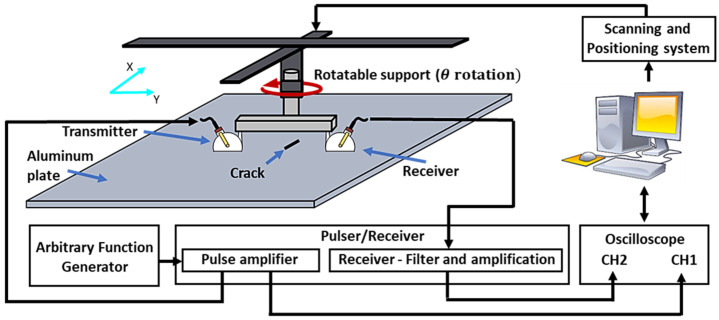
Schematic of experimental setup used for collecting data for Al-1050 specimen study.

The aluminum plate, along with its notch defect, is shown in Figure 1, and the experimental setup of the guided wave measurement system used to study the aluminum plate is shown in the Figure 2.

A pair of transducers (A413S SB at 650 kHz from Olympus) with an angle wedge (ABWX-2001 from Olympus), mounted in a pitch–catch configuration, was used to generate and receive guided wave signals. The width of the wedge is approximately 43 mm. The pair of transducers was attached to a two-axis positioning system with a probe support that allows for the rotation of the two angle beam transducers simultaneously at different angles (*θ*). At each *θ*, line scans of 161 points in steps of 1 mm were performed to collect parallel-beam projection data (B-Scan) in the area of interest. The data were obtained in a 360° range, with intervals of 10°, corresponding to a total of 5796 scanning points. The excitation source was a five-cycle sine burst modulated via a Hann window at 650 kHz. Based on the properties of the dispersion curves in the aluminum plate and the angle beam transducers, the angle of the transmitter was set to 30 degrees to propagate a dominant S0 mode into the plate, in accordance with Snell’s law. Similarly, the angle of the receiver was set to 30 degrees, to receive signals sensitive to the S0 Lamb mode. The excitation signal was generated using an arbitrary waveform generator (AFG 3102) and amplified using a high-power RPR-4000 pulser–receiver instrument. An oscilloscope was used to acquire the excitation signal through channel 1 from a pulse monitor access point located in the pulser/receiver instrument (RPR-4000 from Ritec). The second transducer, acting as the receiver, was connected to the receiver board to amplify and denoise the received signal before acquisition through channel 2 of the oscilloscope. For further information about the scanning system and the scanning parameters, we refer the reader to [13].

### 2.2. Pultruded GFRP Plate

**Figure 3:**
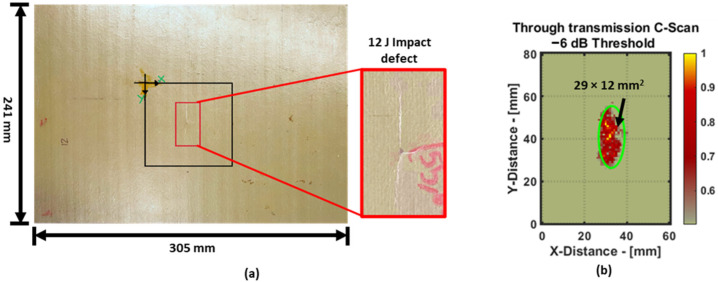
Pultruded GFRP specimen along with 12 J impact damage (**a**) and the ultrasonic air-coupled through-transmission C-Scan image of the impact defect with −6 dB thresholding (**b**).

The second specimen is a pultruded composite GFRP plate made of an E-glass random fiber reinforcement matrix and vinyl ester epoxy resin. The plate was extruded by roving fibers along one direction. An artificial defect was created in the plate via an impact defect with 12 J of energy. Pictures of the plate and the defect are shown in Figure 3a. A C-Scan image of the defect captured via ultrasonic through transmission is shown in Figure 3b. 

The image acquired using the ultrasonic through transmission C-Scan method shows highly detailed information of the defect inside the plate. This plate specimen was previously used in a study [14] to compare the quantification of the impact defect between the through-transmission method and guided wave tomography. However, as for the guided wave tomography results in that article, only one feature, i.e., the amplitude of the signal, was considered as the feature to be used in the reconstruction algorithm. The results from the RAPID damage-imaging algorithm and the defects are quantified in Section 5.3.

**Figure 4:**
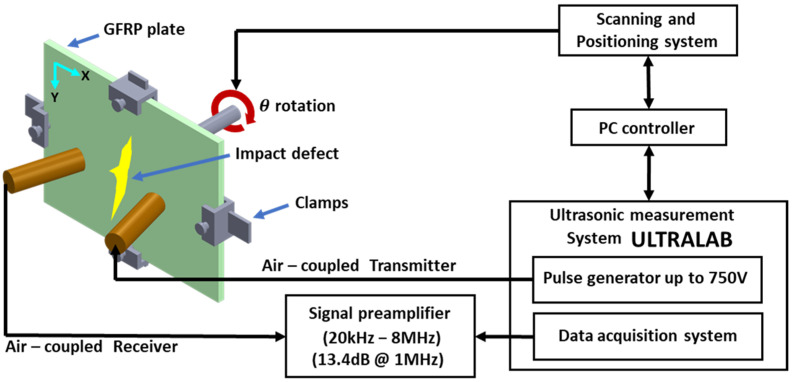
Schematic of air-coupled guided waves used for collecting data in pultruded GFRP plate.

The data for guided wave damage imaging were obtained using 300 kHz unfocused air-coupled transducers. At 300 kHz, the phase velocity of the A0 mode was 1424 m/s. So, according to Snell’s law, the transducers were kept at 14 degrees for the angle of incidence and reception. More details of this air-coupled transducer and its usage for guided waves are explained in [14]. These transducers were controlled using a guided wave measurement system called “Ultralab” developed by the Kaunas University of Technology. The schema of the experimental setup is shown in the Figure 4. The B-Scan in this case was performed with 101 scan points with a 1 mm interval along the y direction. Then, the plate was rotated at a 1-degree angle, and a series of B-Scans were obtained in 360 degrees. This makes a total of 36,360 scanning points.

### 2.3. Laminate GFRP Plate

The third and final specimen is a laminate GFRP made of two types of materials: twill woven fabric WRE581T [0°, 90°] and a biaxial bias stitched fabric XE905 [±45°]. The material properties of the woven and bias fabric are given in Table 2. In total, the laminate GFRP specimen has six layers (woven, bias, and woven)_s_, with a total thickness of the plate of 4 mm.

**Figure 5:**
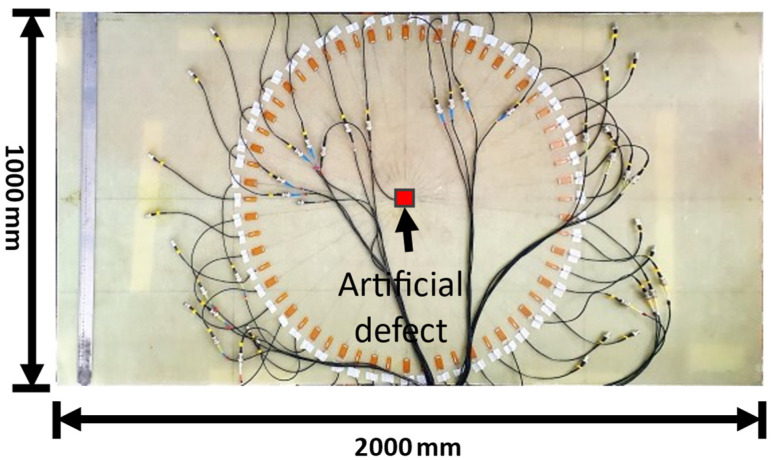
Laminate GFRP specimen used in the study with MFC transducers attached. (On the specimen, 28 mm width transducer, and 12 mm width transducer were placed alternately. But only 28 mm width transducers were considered in this study).

The Macro Fiber Composite (MFC) transducer (M-2814-P1) with dimensions of 28 × 14 mm used in this study was produced by Smart Material GmbH (Dresden, Germany). An excitation frequency of 80 kHz was used for the MFC transducers. A total of 30 of these transducers were fixed on the GFRP plate in a circular array configuration. The transducers were placed at 12° angle increments so that, in total, 30 fan beam projections could be obtained, amounting to a total of 900 scanning points. The composite laminate used for the study is shown in the Figure 5. Using the circular array arrangement, the data for the experiment were acquired via the full matrix capture method using the 128 channel Dasel data acquisition system (Dasel Sistemas, Spain). The defect induced in this experiment was a piece of plexiglass with dimensions of 100 × 100 mm^2^ that was placed at the center of the specimen, and a natural food product, honey, was used as viscoelastic couplant at room temperature (21 °C). This way, a damping effect could be produced, which is like an artificial defect for a guided-wave-based SHM configuration.

## 3. RAPID 

**Figure 6:**
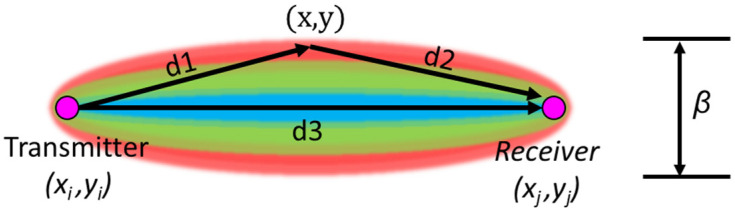
Bayesian probability distribution function used for RAPID algorithm.

RAPID is a technique used for SHM applications that involves the estimation of defect parameters in a structure using Bayesian inference. It is a statistical technique for estimating the probability of an event based on prior knowledge and observed data. One of the advantages of this technique over the tomography method is that the transducers need not be arranged in a perfectly geometric shape like a circle or a rectangle. This method can even be used with an irregular placement of the transducers, making it suitable for monitoring complex geometrical structures. For this method using guided waves, a two-dimensional Bayesian probability distribution function (probability heatmap) was created between each transmitter and receiver path, as shown in Figure 6. 

This probability heatmap *P*(*x*,*y*) is a sum of the product of the Weighted Distribution Function *WDF*(*x*,*y*) and a Damage Index (*DI*) for all transmitter and receiver pairs and can mathematically be written as shown in Equation (1).

(1) $$P\left(x,y\right)=\sum_{i=1}^{N}\sum_{j=1}^{N}{DI}_{ij}\cdot {WDF}_{ij}(x,y)$$

The *WDF* in the spatial domain was constructed using a mesh grid and the two focal points representing the transmitter and receiver locations in the structure. The output of the RAPID algorithm was an image of the structure with a probabilistic location of the defect. In order to obtain this image, the mesh grid was used, and each point in the mesh grid represents a pixel, which can be created using a geometrical function, as in Equation (2)

(2) $${mesh}_{ij}\left(x,y\right)=\frac{d1+d2}{d3}=\frac{\sqrt{{{(x}_{i}-x)}^{2}+{{(y}_{i}-y)}^{2}}+\sqrt{{{(x}_{j}-x)}^{2}+{{(y}_{j}-y)}^{2}}}{\sqrt{{{(x}_{j}-x_{i})}^{2}+{{(y}_{j}-y_{i})}^{2}}}$$

where *d*1 is the Euclidean distance between the transmitter and the mesh grid pixel, *d*2 is the Euclidean distance between the receiver and the mesh grid pixel, and *d*3 is the Euclidean distance between the transmitter and the receiver. The result of this geometrical function resembles an ellipse, as shown in Figure 6, and the size of the ellipse is controlled via the *β* parameter using the Equation (3) called the *WDF*.

(3) $${WDF}_{ij}\left(x,y\right)=\left\{\begin{array}{cc} \frac{\beta -{mesh}_{ij}\left(x,y\right)}{1-\beta }, & if\;\beta >{mesh}_{ij}(x,y)\\ 0, & if\;\beta \leq \;{mesh}_{ij}(x,y) \end{array}\right.$$

The scaling parameter *β* for this algorithm must be greater than 1, and, typically, for a RAPID method from various sources, values of 1.001 and 1.05 are used [6,15] depending on the distance of the transmitter–receiver path. The longer the path, the larger the value of *β* that can be used. Now that the *WDF* has been created for each transmitter–receiver path, this matrix needs to be multiplied by the DI, and then all the resulting matrices must be summed in order to obtain an image of the defect. The DI for this method can be obtained from different signal-processing algorithms, and DIs can be, for example, differential signal amplitude, Time of Flight (ToF), etc. The variety of DI considered for this article is explained in the following section. 

## 4. Features for Reconstruction Algorithm

For tomographic reconstruction, the amplitude or phase is typically used for algorithms in the medical field. But when it comes to guided waves, one can take advantage of the different characteristics of the guided wave signals to obtain various types of DIs (features). The guided waves are usually generated using sine burst in order to avoid multiple wave trains when recording the signal. This way, the recorded signals are more useful for different signal-processing approaches. In this study, the different features of guided wave signals in the time and frequency domains are considered, and they are explained in the following subsections.

### 4.1. Amplitude in Time Domain

**Figure 7:**
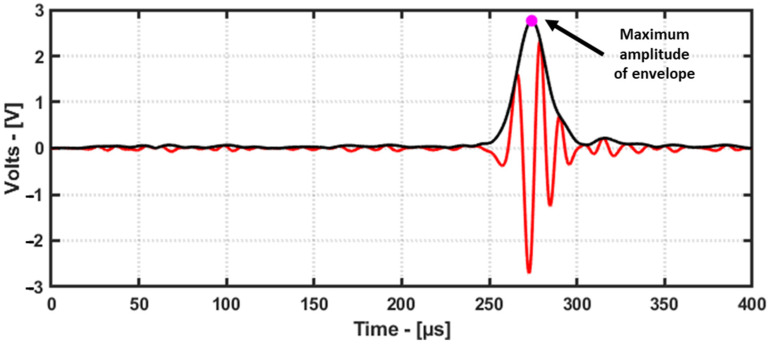
This feature is explained using a signal from laminate GFRP dataset. Maximum amplitude (pink dot) obtained from envelope (black line) of signal (red line) used as feature for damage image reconstruction.

One of the common features used for a reconstruction algorithm is the amplitude of the signal. To obtain the maximum amplitude of the received signals, the envelope of the signal is obtained using the Hilbert transform, and the maximum of the envelope is used as one of the features. This is demonstrated below using a signal from the laminate GFRP specimen (Figure 7).

### 4.2. Deconvolution

**Figure 8:**
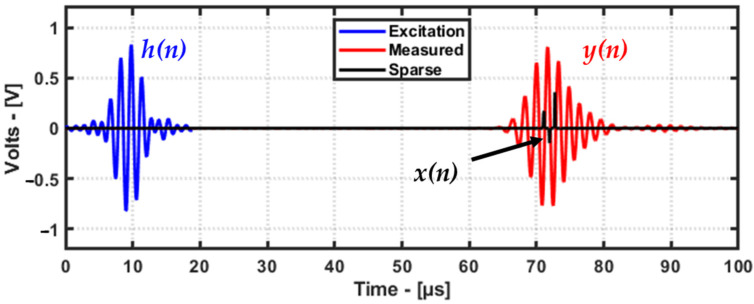
Example of sparse signal obtained after deconvolution; the maximum amplitude of deconvolution was used as a feature for the damage imaging.

Deconvolution is useful in the ultrasonic testing field for increasing the resolution of images obtained from ultrasonic data and for increasing the SNR by denoising the ultrasonic signal. Mathematically, an ultrasonic signal can be written as expressed in Equation (4)

(4) $$y\left(n\right)=\left(h*x\right)\left(n\right)+w\left(n\right)\;\;\;\;\;\;\;\;(\mathrm{volts})$$

where *y*(*n*) is the recorded signal with the ultrasonic sensor. The function *y*(*n*) is the sum of the ambient noise *w*(*n*) and the convolution (the ∗ operator denotes the convolution operation) between the excitation signal *h*(*n*) and the underlying system function *x*(*n*) or sparse signal. Since the excitation signal and the measured signal are known, using the deconvolution method, it is possible to remove the effect of the excitation signal (impulse response) to obtain the sparse signal using an inverse operation. An example of deconvolution for a guided wave signal is demonstrated below using an A-Scan from the aluminum plate dataset (Figure 8).

Since deconvolution is an inverse operation, solving for the sparse signal is conducted via the iterative minimization of the L1 norm cost function. Such a minimization of the cost function in mathematics is called an optimization problem. The reader is referred to [16] for the deconvolution algorithm and the MATLAB code. The optimization formulation for the deconvolution is given in Equation (5), as follows:

(5) $$\arg\underset{x}{min}\frac{1}{2}{\left\|y-Hx\right\|}_{2}^{2}+\lambda {\left\|x\right\|}_{1}$$

Here, *y* is the measured guided wave signal, *H* is the known excitation signal, and *x* is the sparse signal to be identified using deconvolution. The regularization parameter λ and the number of iterations determine how sparse the signal can be. Generally, the λ value is greater than zero. Due to the fact that guided waves can have low SNRs and dispersion due to frequency or changes in material properties, the value of λ and the number of iterations for deconvolution need to be determined in a trial-and-error manner. There are robust deconvolution algorithms such as “SALSA” [17] where parameters such as λ and the number of iterations can be determined in a robust way. 

### 4.3. Reassigned Short-Time Fourier Transform

The Short-Time Fourier Transform (STFT) is a commonly used method for time-frequency analysis that involves dividing a signal into short-time segments and applying a Fourier transform to each segment. The STFT provides a good trade-off between time and frequency resolution but suffers from limitations such as the Heisenberg uncertainty principle and spectral leakage. The “spectrogram” is an extension of the STFT that displays the time-varying spectral content of a signal as a 2D image. The Spectrogram has better time resolution than the STFT but still suffers from the uncertainty principle and resolution limitations.

**Figure 9:**
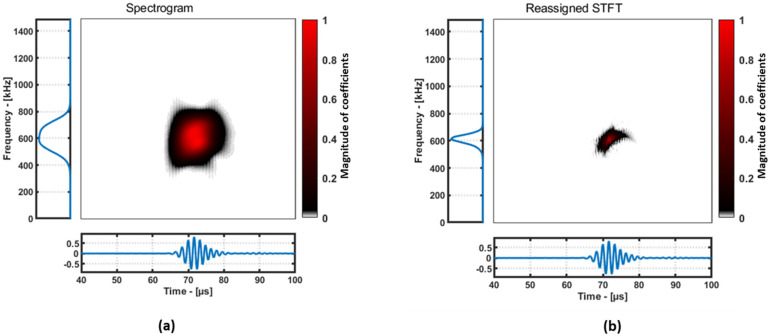
Demonstration of difference between STFT (**a**) and RSTFT (**b**) using a signal.

The Reassigned Short-Time Fourier Transform (RSTFT) is a post-processing technique that improves the time and frequency resolution of the STFT and spectrogram via assigning each time-frequency point to a new location in the time-frequency plane based on its instantaneous frequency and group delay, making it one of the best methods to use for guided wave signals [12], and the code for the RSTFT is available in [18]. An example of a comparison between normal STFT and RSTFT is demonstrated below using an A-Scan from the aluminum plate dataset (Figure 9). For this demonstration, for both the STFT and RSTFT algorithms, a window of 121 samples and an overlap of 120 samples were used. 

**Figure 10:**
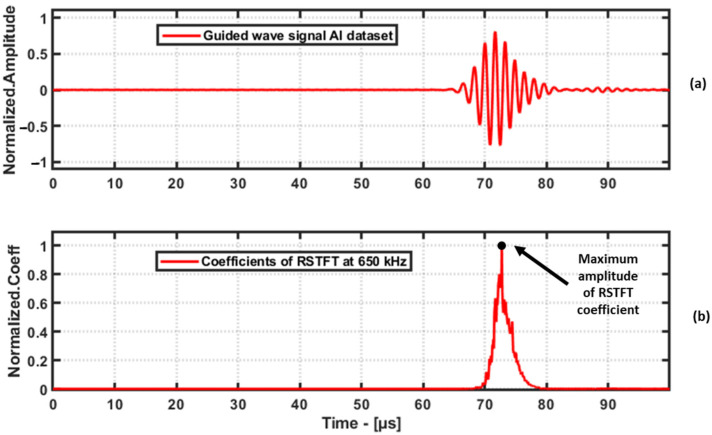
Time domain signal from Al-1050 dataset (**a**) and the RSTFT coefficients at excitation frequency of 650 kHz, (**b**) where the maximum amplitude of the coefficients is taken as the feature for damage imaging.

It is evident from the Figure 9 that the RSTFT performs better than the STFT in terms of localizing the frequencies of the guided waves. After performing the RSTFT, the coefficients along the time axis are extracted at the excitation frequency, and then the maximum amplitude of the coefficients is considered as the feature, and an example of this is shown in Figure 10. The results from RAPID damage imaging using the RSTFT are discussed in Section 5. 

### 4.4. Time of Flight Using Cross-Correlation

**Figure 11:**
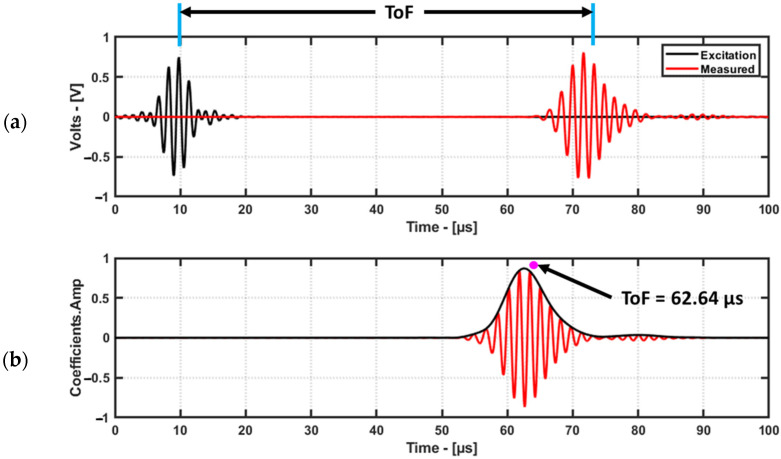
Time domain signal with excitation signal (**a**) and its cross-correlation coefficients with the envelope (**b**). The maximum of the envelope is considered as the feature for damage imaging.

Time of Flight (ToF) can be measured in different ways, such as through cross-correlation, using the envelope from the Hilbert transform [19], and via the zero-crossing technique [20], from the coefficients of the continuous wavelet transform [13]. In this study, cross-correlation was used to determine the ToF as it is one of the simplest and widely used methods for ToF. Cross-correlation was performed between the excitation signal and the measured signal using the MATLAB function “xcorr”. A demonstration of this method using the aluminum dataset is shown in Figure 11.

The resulting coefficients are twice the length of the original signal. So, ignoring the first half of the coefficients will yield coefficients with the same length of the measured signal, which is then plotted vs time. The time index of the envelope maximum of the coefficients will provide the ToF. 

### 4.5. Kurtosis in Time and Frequency Domains

Kurtosis is a statistical measure that quantifies the degree to which a probability distribution’s tails differ relative to the standard Gaussian distribution. In the context of guided wave signals, kurtosis can be used to analyze the shape of the signal in the time and frequency domains. The kurtosis measure is used to assess the non-Gaussian behavior of the received signals, helping to detect and characterize potential defects in the structure. Change in the shape of the received signal envelope, caused by the presence of defects or damage in the path of guided waves, manifests as a variation in the kurtosis value.

In the time domain, a low kurtosis value indicates a flatter distribution, which corresponds to a signal with a wider envelope, and a high kurtosis value indicates a peaked distribution, which corresponds to a signal with a narrow envelope. In general, a change in kurtosis value can indicate the presence of defects or damage in the path of the guided waves in the structure, as these can cause a change in the shape of the signal envelope. Similarly, the kurtosis metric can be applied in the frequency domain as well. However, in the case of the frequency domain, a change in the shape of the frequency spectrum is evaluated.

**Figure 12:**
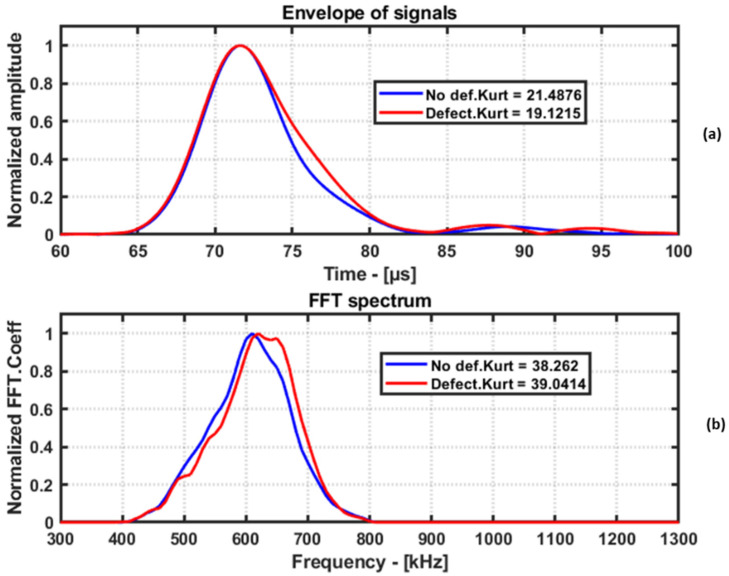
Envelope of no-defect signal and defect signal in time domain (**a**). FFT spectrum of no defect signal and defect signal (**b**). The kurtosis in time domain and FFT domain is used as feature for damage imaging.

A demonstration of kurtosis in the time and frequency domains is shown in Figure 12. The kurtosis value does not change when the amplitudes are scaled. So, in the demonstration, the amplitudes have been normalized to visualize the data in a better way.

## 5. Results

### 5.1. Sinogram Pre-Processing

**Figure 13:**
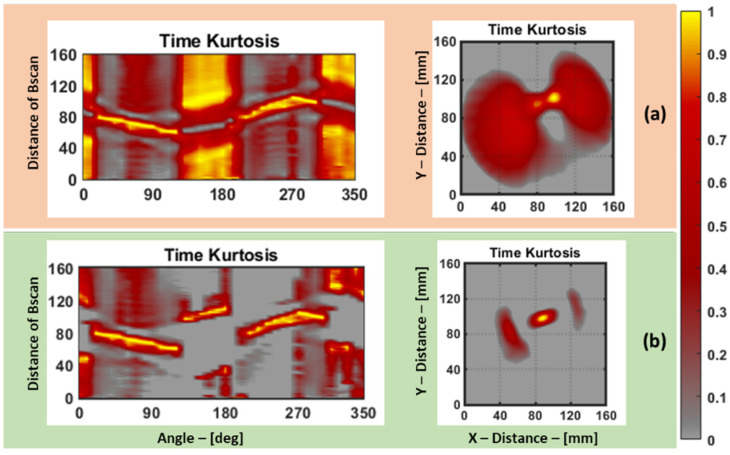
This comparison (**a**,**b**) shows the difference between no pre-processing of the sinogram and its RAPID damage image reconstruction vs. the RAPID damage image reconstruction after pre-processing of the sinogram.

In this context, a sinogram is a matrix of features organized in such a way that each row corresponds to the features extracted from signals in a B-scan projection, while each column signifies the features from subsequent angles in the projections. Now that the features have been extracted from the signals of the three different plates, it is essential to pre-process the data before using them in the damage reconstruction algorithm. In the case of the aluminum plate and the pultruded GFRP plate, the baseline data (data without defects) are not available. In order to eliminate the structural noise surrounding the defect, the sinogram was pre-processed with a few steps, as follows. Firstly, the sinogram was normalized to the range of 0 to 1 so that the highest amplitude in the sinogram represents the defect. Then, the mean value of the sinogram was subtracted, and then the values below 0 in the sinogram were replaced with 0. Then, the sinogram was again normalized to the range of 0 to 1. This way, the value 0 represents the base of the plate, and the values above 0 will mostly represent a defect in the plate. To illustrate the impact of pre-processing, an example is shown in Figure 13. It showcases a comparison between the results obtained without pre-processing and with pre-processing using the sinogram and the damage reconstruction based on the aluminum dataset, specifically focusing on the kurtosis feature in the time domain.

### 5.2. Aluminum Plate

#### 5.2.1. Presentation of the Results

**Figure 14:**
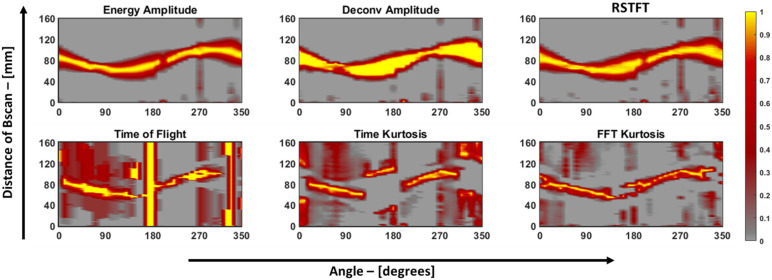
Sinograms of the six features used in this study for aluminum 1050 plate. A value of one indicates a strong presence of damage, and a value of zero represents the base amplitude of the plate, which, in turn, represents the lack of a defect.

**Figure 15:**
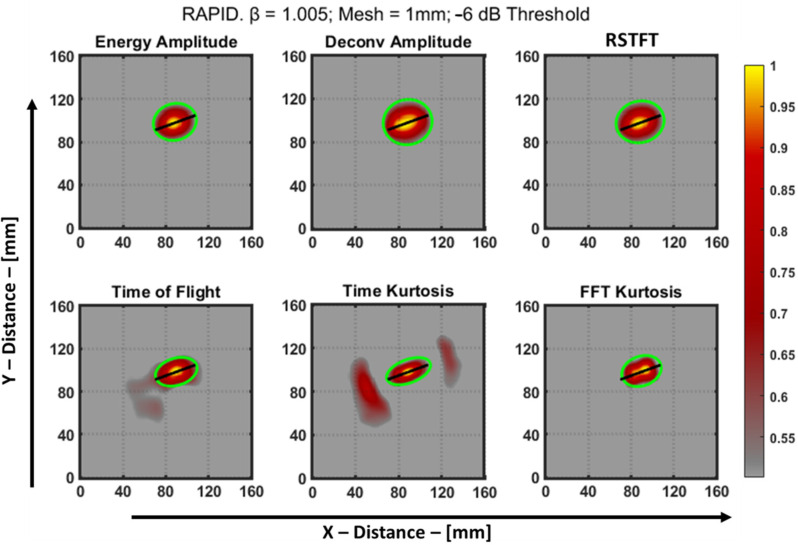
RAPID result of notch type defect from different features of an aluminum plate after −6 dB thresholding. The green ellipse was used to obtain the length, width, and orientation.

The sinograms of the six features of the aluminum plate are shown in Figure 14. Using these sinograms, the RAPID algorithm was run using a *β* value of 1.005, and a grid size of 1 mm was used to acquire a much finer resolution of the image after reconstruction. After normalization, the resulting reconstructed image was then thresholded using a −6 dB method for quantifying the image, and the final defect image is shown in Figure 15. This specimen was previously used by Dario et al. [13] to study the application of a tomographic reconstruction algorithm with different features using guided waves. The authors concluded that only the approximate parameters of a notch-type defect, i.e., the length and orientation of the defect, were observed. So, in the current study, to improve the accuracy of determining the parameters of the defect, features such as those discussed in Section 4 were used for the RAPID algorithm and to quantify the defect.

The sinograms and the reconstructed images are presented with a custom colorbar scale, where values close to 0 are displayed in gray to imitate areas of the aluminum plate where there is no possibility for a defect. The intensity of the defect is represented using a color range from red to yellow. The quantitative green ellipses for the defect were derived by utilizing the “*regionprops*” function in MATLAB. This function enables the calculation of various parameters associated with a defect, including the major axis length, minor axis length, and orientation of the ellipse. These parameters directly correspond to estimates of the length, width, and angle of the notch-type artificial defect present in the aluminum plate. The estimates of the defect are tabulated in Table 3.

#### 5.2.2. Discussion

In a real-world scenario, a notch defect indicates a crack, and it would be possible to determine the length and the orientation of such a crack. Determining the width of a crack is highly dependent on the width of the angle wedge transducer. The width of the angle wedge transducer determines the beamwidth of the guided waves emitted and received. In this case, the width of the angle wedge transducer is 43 mm, which is quite large for identifying the width of the notch. So, in this study, the length of the notch defect was determined using the ToF feature, with a least absolute error of 0.2 mm, and the orientation of the defect was determined using the Kurtosis from the FFT spectrum, with a least absolute error of 0.2 degrees. 

### 5.3. Pultruded GFRP Plate

#### 5.3.1. Display of Results

**Figure 16:**
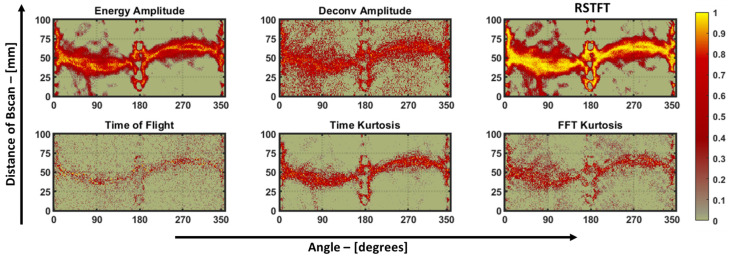
Sinograms of the six features used in this study for pultruded GFRP plate. A value of one indicates a strong presence of damage, and a value of zero represents the base amplitude of the plate, which, in turn, represents the lack of a defect.

**Figure 17:**
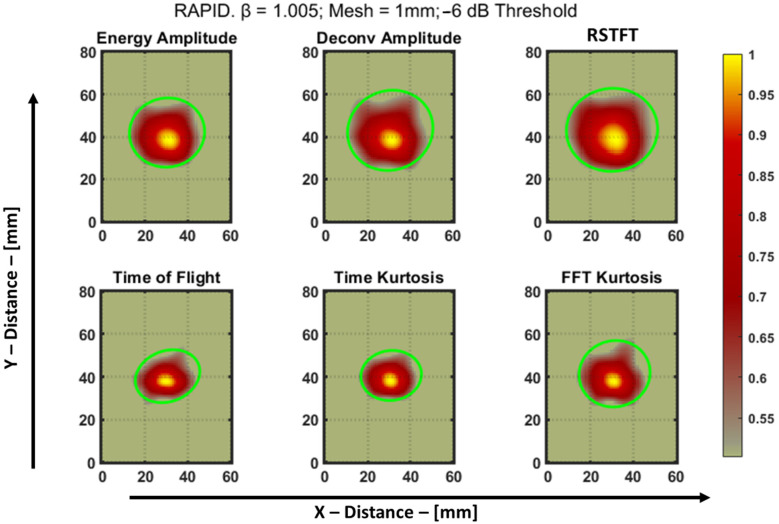
RAPID result of impact type defect from different features in pultruded GFRP specimen after −6 dB thresholding. The green ellipse was used to obtain the length and the width of the defect.

The sinogram data of the pultruded GFRP plate underwent the same processing steps as discussed in Section 5.1, following the methodology employed for the aluminum plate. For the RAPID reconstruction algorithm, a *β* value of 1.005 and a mesh size of 1 mm were used. Subsequently, the ellipse estimation procedure was applied to determine the defect size. In this case, the orientation of the defect holds less significance, as impact damage in composites is typically characterized by the debonding of matrix fibers and delamination effects rather than the notch or crack-type defects observed in metallic plates. Figure 16 displays the sinograms obtained from the six features, and Figure 17 displays the corresponding defect reconstructions. 

To represent the GFRP plate color, values close to 0 were assigned a green color in the figures. Finally, the quantitative results were tabulated in Table 4.

#### 5.3.2. Discussion

In the case of the pultruded GFRP specimen, there are no baseline data (data without a defect). Since only data with defects were available, the sinogram pre-processing was very useful for isolating information about the defect. Moreover, to attain another perspective about the defect, a through-transmission C-Scan was obtained using the same air-coupled transducers at 300 kHz, as shown previously in Figure 3. It should be noted that in the bulk wave through-transmission method, the ultrasonic waves propagate perpendicular to the plate, resulting in a C-Scan image with better resolution. The C-Scan image shows that the impact defect appears more elliptical. However, in the case of guided wave damage reconstruction, the ultrasonic waves propagate inside the plate parallel to its thickness. At the same excitation frequency, such guided wave methods cannot achieve the same level of high-resolution imaging as the through transmission method at 300 kHz. 

Guided waves are generated in the plate structures in circular wavefronts, dispersing the energy in different directions rather than focusing it in one point like in the bulk wave method. Despite this limitation, guided wave damage imaging offers advantages such as rapid coverage of large areas and suitability for single-sided access to a specimen. In guided wave damage imaging, the RAPID algorithm produced a defect image that appeared more circular compared to the through-transmission thickness method. This can be attributed to the fact that guided wave wavefronts are highly sensitive to even the smallest variations in the guided medium. 

Hence, in the case of guided-wave-based damage-imaging methods, the length and width of the impact defect were quantified but not compared with the through transmission method, as shown previously in Table 4. The sinograms of the pultruded GFRP sample appear visually noisier due to the air-coupled transducers used to obtain the data. So, to determine the sinogram with the best SNR, an evaluator called the “Perception-based Image Quality Evaluator” (PIQE) tool available in MATLAB’s image-processing toolbox was employed to analyze the sinograms in block-wise pieces. This evaluator is an unsupervised model capable of quantifying images without relying on any trained data [21]. 

**Figure 18:**
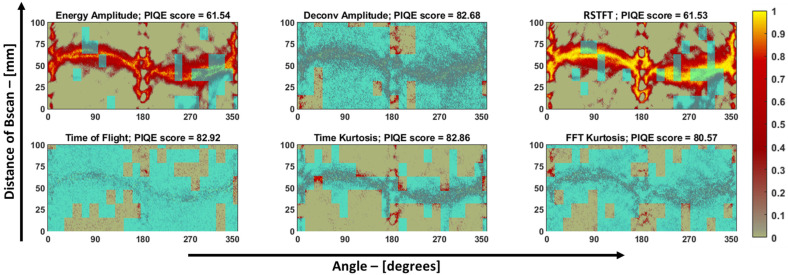
Sinograms of pultruded GFRP specimen from different features. The PIQE mask is overlayed on top of the sinograms in cyan, and the PIQE score is given in the title of each sinogram.

In summary, this tool provides a quantified score in the range of 0–100, representing the image’s noise level. A score of 0 indicates good image quality, while a score of 100 indicates poor image quality. This tool also generates a noise mask for the sinograms. Figure 18 displays the PIQE evaluator’s score and noise mask for each sinogram. Visually, the sinograms obtained from the deconvolution, ToF, kurtosis on time, and FFT features appear grainy (noisy) in comparison to the sinograms obtained from the energy amplitude and RSTFT, which exhibit a higher signal-to-noise ratio. The PIQE score reflects this observation, as the quantified scores for energy amplitude and RSTFT are low in comparison to the other features, and this trend was supported when using the PIQE masks as well.

### 5.4. Laminate GFRP Plate

In SHM, particularly when utilizing guided waves, a common approach involves comparing baseline signals (data without defects) with signals acquired after a specific time or multiple loading/unloading cycles of the structure. By making this comparison, any changes in the signals can indicate the presence of defects or degradation of elastic properties. In the case of the laminate GFRP specimen, baseline data are available; this differs from the case for the previous two specimens, where baseline data were not accessible. In those cases, the sinogram pre-processing proved useful in isolating information about the defects. However, with the laminate GFRP data, the concept of differential imaging can be employed to obtain an image of a defect. The differential damage imaging in the laminate GFRP was demonstrated using the energy amplitude feature. 

**Figure 19:**
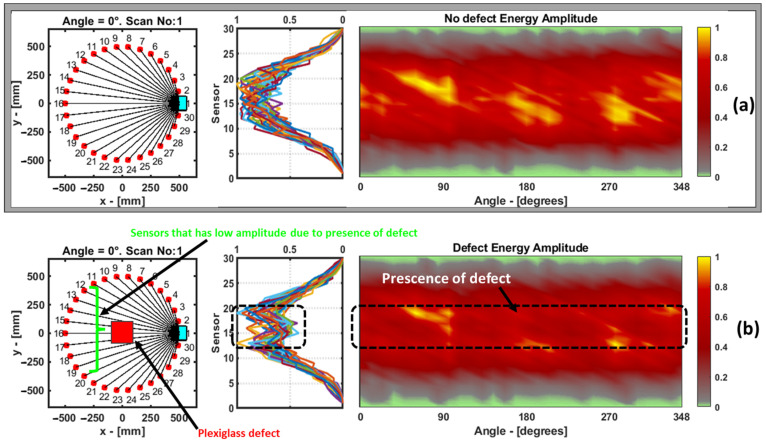
Sinogram of data without defect (**a**) and with defect (**b**) from energy amplitude feature in laminate GFRP sample. To the left of the sinogram is the plot with all the projections. Each colored line represents a feature from each particular projection.

In this case, both the sinograms from the dataset without the defect and the dataset with the defect were normalized from zero to one, as shown in Figure 19, and for the case of the sinogram with the defect, the presence of the defect was very subtle and not easy to identify. On the left side of the sinogram, a plot is shown, where each colored line represents the amplitude of the features from one projection at a particular angle. Since the plexiglass defect is in the center of the transducer circle, the transducers from 12 to 19 show slightly lower amplitudes. 

**Figure 20:**
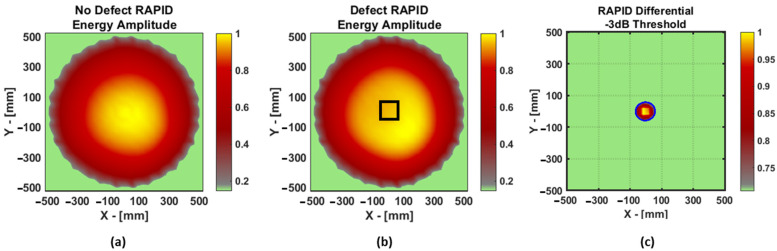
Laminate GFRP. RAPID result for data without defect (**a**) and with defect (**b**) and the differential image after −3 dB thresholding (**c**). In the case of (**b**,**c**), the location of the defect is indicated by the black square in the middle. In the case of (**c**), the ellipse estimated after thresholding is depicted in blue.

Then, the RAPID algorithm was executed for both the datasets, as shown in Figure 20. In these images, the presence of the defect is even more difficult to identify, but upon performing differential image and −3 dB thresholding, the defect could be identified and then quantified using the ellipse method. The width (x axis) and the length (y axis) of the defect were found to be 115.18 mm and 123.18 mm, respectively, while the original dimensions of the defect were 100 × 100 mm^2^. 

## 6. Conclusions

This study represents a significant advancement in assessing the efficacy of the RAPID algorithm with diverse features such as Damage Indices (DIs) across various damage scenarios and sample types. It serves as a critical step in comprehensively evaluating this algorithm’s performance. To achieve this, diverse features were extracted and analyzed from guided wave signals originating from three distinct specimens: aluminum, pultruded GFRP, and laminated GFRP. The extracted features were the amplitude of the sparse signal, the amplitude of the coefficients at the excitation frequency from the RSTFT, the ToF obtained from cross-correlation, kurtosis in the time domain, and kurtosis in the frequency domain. Subsequently, the RAPID algorithm was employed to assess damage-imaging performance using these features.

In case of the notch-type defect in the aluminum plate, it was observed that the ToF feature provided a more accurate estimation of defect length, while the kurtosis feature extracted from the FFT spectrum yielded a superior estimation of defect orientation. In cases where the actual dimensions of damage are unknown, such as the case for the second specimen (pultruded GFRP), evaluators like PIQE can be employed to evaluate the quality of feature sinograms. The authors recommend using the ToF feature only in cases where the distance between the transducer and the receiver is the same, i.e., in the case of the first two methods in this study, where the parallel beam guided waves were employed to collect the data. The other features can be tested for different situations. As demonstrated in this study, it is necessary to explore different features, as the RAPID algorithm can perform better with different features. But it is not possible to conclude that one feature is the best feature for this specific material (for example, laminate GFRP, pultruded GFRP, etc.). Such a decision can only be made by considering the type of the defect and many other parameters for a real-world problem. But the proposed approach presented in this study can be implemented to evaluate relevant features for damage imaging in different kinds of materials.

The practical usage of the methodology proposed in this study depends on the specific application. When conducting damage inspections with guided waves, whether during the annual inspection of aircraft to detect subsurface cracks or in the manual inspection of wind turbines for assessing impact defects like those caused by bird or lightning strikes, one can employ the parallel-beam guided wave inspection method. As illustrated for the first two specimens in this study, this approach enables the evaluation of structure using the baseline-free RAPID algorithm. Moreover, in the first two specimens, the quantities of scanning points were 5796 and 36,360, respectively, which are relatively large when compared to the 900 coarse scanning points for the laminate GFRP specimen. This can be challenging with respect to implementing a baseline-free methodology in some cases because the difference in amplitude between the defect-free and defect specimen may not be significant enough to differentiate between the two situations. So, for SHM configurations like the third specimen in this study, a differential-imaging-based RAPID method becomes much more suitable.

The methodologies employed in this investigation were executed within an ex situ laboratory setting and by considering artificial defect conditions. Presently, another examination is in progress, focusing on the application of these methodologies to an aircraft wing structure constructed from carbon fiber composite material. Notably, this wing specimen presents a heightened level of complexity due to factors such as asymmetrical sensor placement and the presence of structural stiffeners. The outcomes of this ongoing research will be published in a forthcoming scientific article.

## Figures and Tables

**Table 1:** Material and defect description.

Material and Dimensions	Transducer Used	Elastic Properties	Defect Type and Parameters
Isotropic Aluminum plate of 1050 grade. 500 × 500 × 2 mm^3^	Contact-type angle wedge transducer	*E* = 71 GPa *ν* = 0.33 *ρ* = 2705 kg/m^3^	Notch-type defect. 40 × 2 × 1 mm^3^ Orientation: 20°
Quasi-isotropic pultruded GFRP plate. 305 × 241 × 3.2 mm^3^	Non-contact-type air-coupled transducer	*E* = 10.5 GPa *ν* = 0.36 *ρ* = 1342 kg/m^3^	12 J impact defect. 33 × 10 mm^2^
Quasi-isotropic laminate GFRP plate. 2000 × 1000 × 4 mm^3^	Contact-type Macro Fiber Composite interdigitated transducer	Described in Section 2.3	Artificial defect. Square-shaped Plexiglass. 100 × 100 mm^2^

*E* = Young’s Modulus; *ν* = Poisson ratio; and *ρ* = Density.

**Table 2:** Properties of laminate GFRP plate.

Properties	WRE581T	XE905	Units
Volume fraction (*V_f_*)	45	46	%
Young’s modulus (*E*_1_ = *E*_2_)	20.38	21.22	GPa
In-plane shear modulus (*G*_12_)	3.28	3.05	GPa
Interlaminar shear modulus (*G*_23_)	2.98	3.05	GPa
Poisson’s ratio (*ν*_12_ = *ν*_23_)	0.12	0.12	-
Density (*ρ*)	1778	1786	kg/m^3^
Ply weight	881	1364	g/m^2^
Structural thickness	0.5	0.75	mm

**Table 3:** Quantitative estimates of notch parameters in Al-1050 specimen obtained from RAPID damage-imaging algorithm.

Feature	Notch Width (mm)			Notch Length (mm)			Orientation (Degrees)		
	Real	Estimate	Abs Error	Real	Estimate	Abs Error	Real	Estimate	Abs Error
Amplitude	2	33.99	31.99	40	40.51	0.51	20	15.7	4.3
Deconvolution	2	41.61	39.61	40	45.46	5.46	20	13.8	6.2
RSTFT	2	38.49	36.49	40	45.13	5.13	20	14.7	5.3
Time of flight	2	25.19	23.19	40	39.8	0.2	20	17	3
Time kurtosis	2	21.49	19.49	40	42.86	2.86	20	20.8	0.8
Frequency kurtosis	2	27.42	25.42	40	37.85	2.15	20	20.2	0.2

**Table 4:** Quantitative estimates of the impact defect in pultruded GFRP specimen obtained from RAPID damage-imaging algorithm.

Feature	Width (mm) (X Length)	Height (mm) (Y Length)
Amplitude	32.53	35.49
Deconvolution	37.28	40.71
RSTFT	39.07	43.02
Time of flight	23.66	31.03
Time kurtosis	23.28	28.44
Frequency kurtosis	30.87	33.41

## Data Availability

The data presented in this study are available on request from the corresponding author.

## References

[1] Michaels J.E. (2008). Detection, Localization and Characterization of Damage in Plates with an In Situ Array of Spatially Distributed Ultrasonic Sensors. Smart Mater. Struct..

[2] Nokhbatolfoghahai A., Navazi H.M., Groves R.M. (2021). Evaluation of the Sparse Reconstruction and the Delay-and-Sum Damage Imaging Methods for Structural Health Monitoring under Different Environmental and Operational Conditions. Measurement.

[3] Eremin A., Glushkov E., Glushkova N., Lammering R. (2019). Guided Wave Time-Reversal Imaging of Macroscopic Localized Inhomogeneities in Anisotropic Composites. Struct. Health Monit..

[4] Agrahari J.K., Kapuria S. (2018). Active Detection of Block Mass and Notch-Type Damages in Metallic Plates Using a Refined Time-Reversed Lamb Wave Technique. Struct. Control Health Monit..

[5] Albiruni F., Cho Y., Lee J.H., Ahn B.Y. (2012). Non-Contact Guided Waves Tomographic Imaging of Plate-like Structures Using a Probabilistic Algorithm. Mater. Trans..

[6] Wang S., Wu W., Shen Y., Liu Y., Jiang S. (2020). Influence of the Pzt Sensor Array Configuration on Lamb Wave Tomography Imaging with the Rapid Algorithm for Hole and Crack Detection. Sensors.

[7] Herrera R.H., Liu Z., Raffa N., Christensen P., Elvers A. (2015). Improving Time Estimation by Blind Deconvolution: With Applications to TOFD and Backscatter Sizing. arXiv.

[8] Chapon A., Pereira D., Toews M., Belanger P. (2021). Deconvolution of Ultrasonic Signals Using a Convolutional Neural Network. Ultrasonics.

[9] Herrera R.H., Orozco R., Rodriguez M. (2006). Wavelet-Based Deconvolution of Ultrasonic Signals in Nondestructive Evaluation. J. Zhejiang Univ. Sci..

[10] Honarvar F., Sheikhzadeh H., Moles M., Sinclair A.N. (2004). Improving the Time-Resolution and Signal-to-Noise Ratio of Ultrasonic NDE Signals. Ultrasonics.

[11] Mirahmadi S.J., Honarvar F. (2011). Application of Signal Processing Techniques to Ultrasonic Testing of Plates by S0 Lamb Wave Mode. NDT E Int..

[12] Niethammer M., Jacobs L.J., Qu J., Jarzynski J. (2000). Time-Frequency Representation of Lamb Waves Using the Reassigned Spectrogram. J. Acoust. Soc. Am..

[13] Pasadas D.J., Barzegar M., Ribeiro A.L., Ramos H.G. Guided Lamb Wave Tomography Using Angle Beam Transducers and Inverse Radon Transform for Crack Image Reconstruction. Proceedings of the Conference Record—IEEE Instrumentation and Measurement Technology Conference.

[14] Asokkumar A., Jasiūnienė E., Raišutis R., Kažys R.J. (2021). Comparison of Ultrasonic Non-contact Air-coupled Techniques for Characterization of Impact-type Defects in Pultruded Gfrp Composites. Materials.

[15] Sheen B., Cho Y. (2012). A Study on Quantitative Lamb Wave Tomogram via Modified RAPID Algorithm with Shape Factor Optimization. Int. J. Precis. Eng. Manuf..

[16] Ivan Selesnick Sparse Deconvolution (An MM Algorithm). https://cnx.org/contents/8nON5rNt@5/Sparse-Deconvolution-An-MM-Algorithm.

[17] Chang Y., Zi Y., Zhao J., Yang Z., He W., Sun H. (2017). An Adaptive Sparse Deconvolution Method for Distinguishing the Overlapping Echoes of Ultrasonic Guided Waves for Pipeline Crack Inspection. Meas. Sci. Technol..

[18] Mariia Fedotenkova Spectrogram Reassignment. https://github.com/mfedoten/reasspectro.

[19] Xu B., Yu L., Giurgiutiu V. (2021). Advanced Methods for Time-of-Fiight Estimation with Application to Lamb Wave Structural Health Monitoring. Structural Health Monitoring 2009: From System Integration to Autonomous Systems, Proceedings of the 7th International Workshop on Structural Health Monitoring, IWSHM 2009, Stanford, CA, USA, 7–9 December 2021.

[20] Draudviliene L., Meskuotiene A., Mazeika L., Raisutis R. (2017). Assessment of Quantitative and Qualitative Characteristics of Ultrasonic Guided Wave Phase Velocity Measurement Technique. J. Nondestruct. Eval..

[21] Venkatanath N., Praneeth D., Maruthi Chandrasekhar B.H., Channappayya S.S., Medasani S.S. Blind Image Quality Evaluation Using Perception Based Features. Proceedings of the 2015 21st National Conference on Communications, NCC 2015.

